# Female Urethral Adenocarcinoma Posing a Diagnostic Challenge

**DOI:** 10.1002/ccr3.70716

**Published:** 2025-07-30

**Authors:** Omar Darboe, Bartholomeo Nicholaus Ngowi, Gideon Mwasakyalo, Christine Mwakio, Rosemary Wangari Kamau, Adan S. Bashir, Waithera David, Paul Juma Irungu

**Affiliations:** ^1^ Department of Urology Kilimanjaro Christian Medical Centre Moshi Tanzania; ^2^ Department of Oncology Kijabe Kijabe Kenya; ^3^ Department of Pathology Kijabe Hospital Kijabe Kenya; ^4^ Department of Urology Kijabe Kijabe Kenya

**Keywords:** argon plasma coagulation, chemoradiotherapy, female urethral adenocarcinoma, multidisciplinary team, radiation proctitis

## Abstract

Generally, urethral malignancies are uncommon urological tumors that are more frequent in women than in men. The etiology of female urethral adenocarcinoma is yet unknown. Recurrent UTIs and urethral diverticula are two common risk factors linked to it. Clinical presentations can differ and are not always specific. A complete history and physical examination are part of management; urethrocystoscopy with urethral biopsy is used for diagnosis; investigations such as magnetic resonance imaging or abdominal computed tomography scans are used for staging; and treatment options include monotherapy and multimodal therapy. A 53‐year‐old female who presented with a 9‐month history of lower urinary tract symptoms characterized by a burning sensation when passing urine, increased urinary frequency, and a feeling of incomplete bladder emptying associated with a feeling of a vaginal mass. A fixed fungating mass at the external urethral orifice was seen. Pelvic magnetic resonance imaging reported a retropubic urethral mass with bilateral inguinal lymph nodes. She underwent urethrocystoscopy with multiple urethral biopsies taken, and the specimen was sent for histopathology, which confirmed a well‐differentiated urethral adenocarcinoma with mucin production. She was treated with chemoradiotherapy as per multidisciplinary team (MDT) meeting recommendations. She developed symptoms of radiation proctitis, which were treated with argon plasma coagulation. She has since been on follow‐up at our oncology clinic, and a subsequent positron emission tomography CT scan reported no tumor recurrence or metastasis. She is currently doing well with no recurrence of symptoms. Although female urethral adenocarcinoma is uncommon, it requires comprehensive investigation when it is suspected, particularly in women who have nonspecific or recurrent lower urinary tract symptoms. Management should involve a MDT approach where available. Depending on the disease's stage and location, treatment options may include surgery, nonsurgical options such as radiotherapy and chemotherapy, or a combination. Patients should be monitored for any signs of recurrence of the illness. A better prognosis is said to exist for distal urethral cancers that are localized.

**Summary**

- Urethral Adenocarcinoma is an uncommon urological malignancy with a higher preponderance in women than in men.
- It often presents with nonspecific symptoms. There is a need for proper history, thorough examination, and MDT management, with multimodal therapy being very effective when done properly.
- Patients should be monitored for any signs of recurrence of the illness after initial treatment.

## 1 Introduction

Globally, about 0.02% of all female cancers and fewer than 1% of female genitourinary tract tumors are female urethral carcinomas. The histological variation of this rare disease includes 70% of cases of squamous cell carcinoma, 20% of transitional cell carcinomas, and 8%–10% of adenocarcinomas. Primary urethral adenocarcinoma is more common in females than in males and is thought to arise from the skene's glad [1]. In Kenya, where this case was managed, data on female urethral adenocarcinoma is limited. Most studies done and cited were on other genitourinary tumors. WHO urethral cancer classification Urethral adenocarcinoma in females is further subcategorized into two histological variants, which are mucinous/columnar and clear cell types [2]. Neuroendocrine tumors, lymphoma, urethral diverticular, vulvar or vaginal melanoma, urethral polyps, caruncle, and leiomyoma are among the common differentials of urethral adenocarcinoma [3, 4].

Urinary frequency, localized tenderness, urethrorrhagia, urethral obstruction, dysuria, urinary tract infection, concomitant hematuria or urethrocutaneous fistula, and dyspareunia are examples of clinical symptoms that are neither pathognomonic nor diagnostic for this disorder. Our patient presented with some of these symptoms which include increased urinary frequency, dysuria, recurrent urinary tract infections, sensation of incomplete bladder emptying, which was associated with feeling of a vaginal mass and foul‐smelling vaginal discharge. These nonspecific symptoms cause delay in diagnosis and lead to more advanced diseases. The clear cell histological subtype has been linked to congenital associations with recurrent urinary tract infections and urethral diverticula as risk factors.

A complete history, physical examination, and diagnostic tests like urethrocystoscopy and abdominal CT or MRI are all part of the diagnosis process. The diagnosis is confirmed by a urethral biopsy. The prognosis is dependent on the disease's location, histological grade, clinical stage, and treatment options, which include monotherapy or multimodal therapy [5, 6].

In this paper, we discuss the case of a 53‐year‐old female who was received at Africa Inland Church (AIC) Kijabe Hospital in Kenya from the gynecology clinic of the same hospital. She was diagnosed with urethral adenocarcinoma with mucin production confirmed by histopathology and managed accordingly. The aim of writing this case is to highlight the importance of thorough assessment of female patients presenting with a urethral mass, involving other concerned specialties early when in doubt and the role of early multidisciplinary team (MDT) management in patients presenting with urethral adenocarcinoma. It is also to highlight the importance of lower‐level service provision centers to refer patients presenting with recurrent urinary tract infections rather than managing them repeatedly for the same diagnosis.

## 2 Case Management

### 2.1 History

The case is a 53‐year‐old female who presented with a 9‐month history of lower urinary tract symptoms characterized by dysuria, increased urinary frequency, and a feeling of incomplete bladder emptying, which was associated with a feeling of a vaginal mass, foul‐smelling vaginal discharge, and denied bleeding per vagina or dyspareunia. Health care infrastructure in Kenya is structured from Levels 1 to 6. Levels 1–3 involve community health services, dispensaries, clinics, and health centers, and service provision is by nurses and clinical officers. Levels 4–6 involve subcounty hospitals, county referral hospitals, and national referral hospitals, respectively, where all cadres provide services. This patient was seen at a Level 3 facility where service is mainly provided by clinical officers and nurses. She was treated for recurrent urinary tract symptoms for a period of 6 months on periodic antibiotics. She had a history of total abdominal hysterectomy 7 years prior to her presentation to us secondary to multiple symptomatic fibroids; the pap smear was negative. She is a P4 + 0, all alive; the last born is 15 years. She is a secondary smoker.

### 2.2 Examination

**FIGURE 1:**
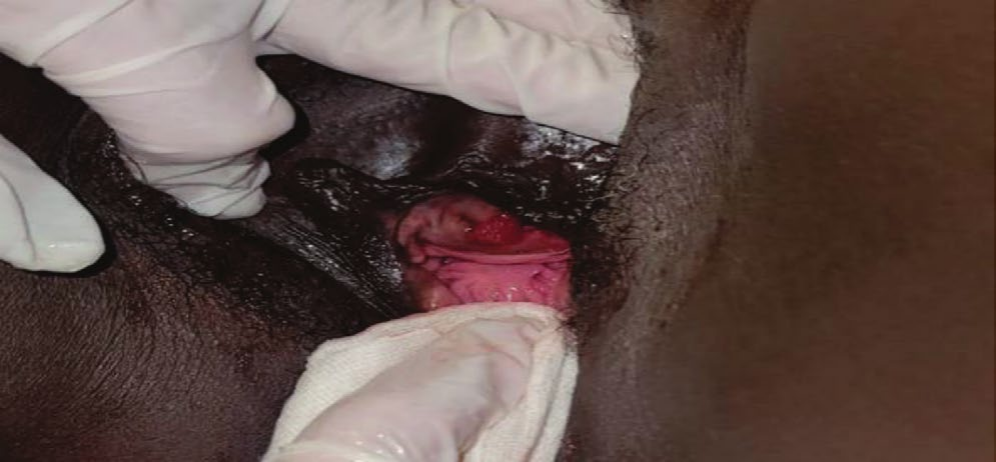
Image showing an inferior‐lateral fungating urethral meatal lesion.

Examination was done by a consultant Urologist; general examination was unremarkable. Focal examination revealed a protruding 6 cm × 5 cm fungating mass at the urethral orifice that was fixed, tender, with associated contact bleeding at the inferior aspect, and palpable bilateral inguinal lymph nodes (Figure 1).

Examination under anesthesia and urethrocystoscopy with urethral biopsy was done by the consultant Urologist at AIC Kijabe Hospital. Intraoperative findings were a fungating urethral orifice mass and a panurethral mass extending close to the bladder neck. The bladder mucosa was normal. Several biopsy specimens were taken from the urethra by the Urologist and sent for histopathology.

### 2.3 Investigations

**FIGURE 2:**
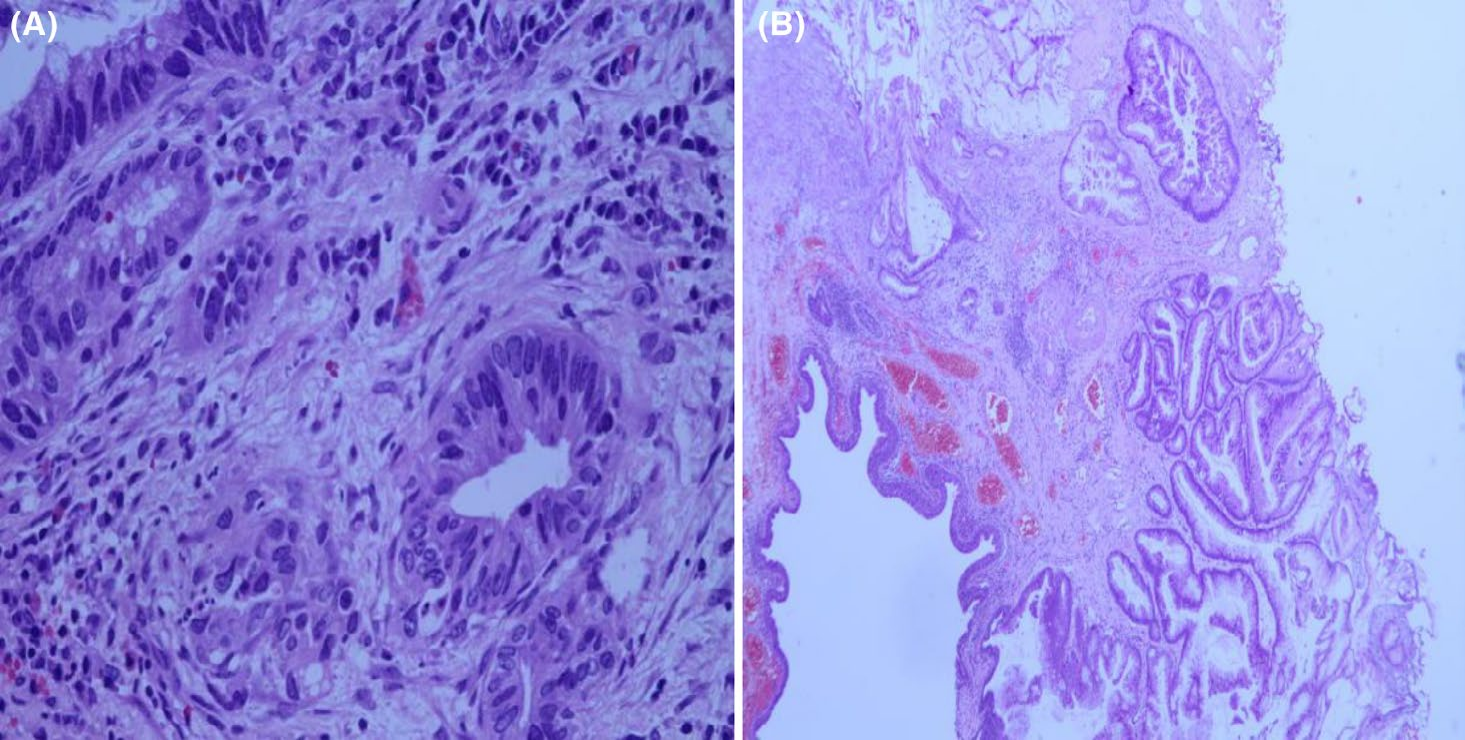
Showing histology slides of the urethral adenocarcinoma with (A) showing a gland with cells arranged in irregular patter and (B) showing finger‐like projections of the urothelial lining, blood vessels, and area of mucin production.

The specimen was processed and analyzed by the consultant pathologist at AIC Kijabe hospital and reported as a well‐differentiated urethral adenocarcinoma with mucin production; a gland with cells arranged in an irregular pattern on (Figure 2A,B) showing finger‐like projections of the urothelial lining, blood vessels, and an area of mucin production.

**FIGURE 3:**
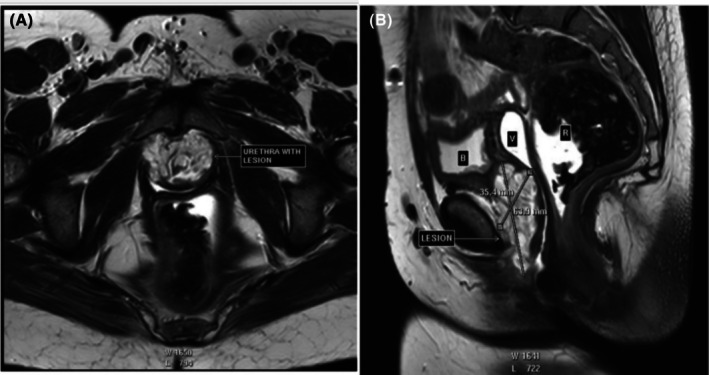
(A) Showing MRI images of the urethral lesion and (B) the surrounding pelvic organs.

After a thorough history was taken, examination was done and biopsy of the lesion taken, a Pelvic magnetic resonance imaging (MRI) was requested by the consultant urologist as the gold standard imaging. The aim was to establish the local extent of the tumor, presence of metastasis as well as pelvic lymph node involvement. The MRI was reported by the consultant radiologist at AIC Kijabe Hospital and reported a cauliflower‐like mass (Figure 3A) involving the entire retropubic urethra measuring 63.9 × 35.4 × 45.0 mm, invading the endopelvic fascia, lower urinary bladder neck, pubourethral, periurethral, paraurethral ligaments bilaterally, perineal membrane, and clitoral hood with a TNM staging of T4N2Mx (Figure 3B).

### 2.4 Treatment

The case was then discussed by an oncologist, radiologist, pathologist, and urologist at a MDT meeting at AIC Kijabe Hospital; a decision was made to perform a staging positron emission tomography (PET) scan first, which revealed an ill‐defined hypodense retropubic urethral mass that measured 62 × 39 × 41 mm and was consistent with an already‐known primary tumor.

Chemoradiation therapy as treatment was initiated after MDT consultation. This was administered by the oncologist. She received 28 cycles of radiotherapy and 6 cycles of chemotherapy.

### 2.5 Follow‐Up

Three months after completing her treatment, she presented with complaints of pain on passing stool, perineal skin lesions that were itchy, and an inability to sit down. Examination revealed perianal skin desquamation extending to the vulva, and an assessment of radiation proctitis was made. She was again discussed with the MDT, and a decision was made to send her for argon plasma coagulation.

## 3 Discussion

Female urethral adenocarcinoma is a rare urological malignancy; in this paper, we discuss the case of a 53‐year‐old female who was managed with chemoradiation in which we noted the importance of early diagnosis and a multidisciplinary approach to be beneficial. Although the exact cause of female urethral adenocarcinoma is unknown, literature revealed associations of the tumor with the paraurethral skene's gland, which is similar to the prostate gland; consequently, PSA staining may be present in these tumors; however, this may not be the case in most cases. Different studies on histological subtype prevalence showed varying results; it is found that the higher incidence of this condition is in those above 50 years [6, 7].

The most common etiological risk factors reported are recurrent urinary tract infections and urethral diverticula; however, different studies have mentioned other factors such as leukoplakia, chronic irritation, human papilloma virus infection or other viral infections, polyps, caruncles, chronic irritation due to indwelling catheters, and sexually transmitted diseases. Some reported the congenital association in the case of the clear cell subtype [8, 9]. Carvalho Neto and colleagues in 2016 [1] hypothesized an association between smoking and urethral adenocarcinoma. In addition to being treated for recurrent urinary tract infections, our patient also smoked as a secondary smoker; her spouse was a chain smoker.

Clinical symptoms may vary depending on the timing of presentation but can be either obstructive lower urinary tract symptoms (straining and feeling of incomplete bladder emptying) or irritative (increased urinary frequency and dysuria) or both, hematuria or bloody urethral discharge, and a protruding urethral mass. There may be associated dyspareunia, abscess, or urethrocutaneous fistula in locally advanced cases [5, 10]. Our patient had both obstructive and irritative symptoms with a fungating urethral mass, associated foul‐smelling vaginal discharge, and contact bleeding on examination.

A complete history, physical examination, examination under anesthesia, and serum workups such as a complete blood count, serum creatinine and urea, urinalysis, and urine cytology are all part of the patient assessment and workup process. Abdominal‐pelvic MRI, CT, and ultrasonography scans can all be performed; however, MRI is the preferred method for determining the location, size, and local infiltration of the tumor. A CT scan of the abdomen, chest, and pelvis is recommended for staging with pre‐ and post‐contrast images for assessment of the entire urinary tract [11].

A cystoscopy with urethral tumor biopsy is diagnostic [2, 12]. A PET CT scan can also be used for staging workup, as done in our case if available.

Tumor spread can be local or distant metastasis. In local infiltration, it more commonly spreads to the vagina, urinary bladder, or other periurethral tissues, whereas in distant metastasis, it spreads more commonly to the lungs, liver, bone, and brain [2, 13].

In our case, there was a tumor spread to the bladder neck and periurethral tissue with inguinal lymphadenopathy.

Marital status should be flagged as it was shown that unmarried women tend to present with more locally advanced diseases and unmarried men do not benefit from multimodal therapy, though both groups require pre‐ and post‐treatment counseling and follow‐up. Furthermore, Morra and colleagues noted that there was lower cancer‐specific survival in married women in comparison to married men [14, 15].

Management of female urethral adenocarcinoma depends on several factors, such as the stage of the tumor, the histological grade, as well as the location. It can be monotherapy or multimodal therapy [16]. Distal focal tumors tend to have a better prognosis than proximal infiltrating or distant metastatic tumors. In the surgical management of proximal female urethral adenocarcinoma, a complete or partial urethrectomy can be done. In distal tumors, a urethra‐sparing surgery, urethrectomy, or radiotherapy can be done, and the patient should be followed up for possible salvage surgery or radiotherapy in case of recurrence. In advanced tumors, neoadjuvant chemotherapy and surgery or chemoradiotherapy or surgery and adjuvant radiotherapy can be done. The decision to do chemotherapy is best advised after a MDT discussion [17, 18].

Junjie et al. [9], however, reported that in their second case in the paper they published, the patient was not routinely followed up following surgical resection. Additionally, the patient refused adjuvant multimodal therapy and eventually developed distant metastasis, necessitating palliative care, even after the tumor recurrence was discovered a few years later with a second surgery performed. This highlights that in cases of systemic or locally advanced disease, multimodal therapy is necessary, as is routine patient follow‐up to ensure the best possible care. In our patient with a locally invasive disease, a chemoradiotherapy model of treatment was used, and the patient had radiation proctitis as a complication, as reported by [11].

Chemotherapy has shown survival benefits in adenocarcinoma and other variants of urethral cancers but not in squamous cell carcinoma. Unconventional nonmetastatic urethral malignancy histology harbors 106‐month survival benefit, while the metastatic unconventional has only 10 months [18, 19].

## 4 Conclusions

Female urethral adenocarcinoma being uncommon, it requires a comprehensive investigation when it is suspected, particularly in those who have nonspecific or recurrent lower urinary tract symptoms. MRI is the ideal imaging method for determining the location, size, and local infiltration of the tumor. Management should involve a MDT. Monotherapy or multimodal therapy can be used depending on the disease's location, histological grade, and clinical stage. Localized distal urethral cancers are said to have a better prognosis. Follow‐up is paramount to monitor recurrence.

## Author Contributions

**Omar Darboe:** conceptualization, writing – original draft. **Bartholomeo Nicholaus Ngowi:** writing – review and editing. **Gideon Mwasakyalo:** writing – review and editing. **Christine Mwakio:** formal analysis. **Rosemary Wangari Kamau:** formal analysis. **Adan S. Bashir:** writing – review and editing. **Waithera David:** writing – review and editing. **Paul Juma Irungu:** writing – review and editing.

## Consent

A written informed consent was obtained for the patient's clinical information to remain anonymous.

## Conflicts of Interest

The authors declare no conflicts of interest.

## Data Availability

Data are available to the corresponding author upon request.
